# Oral Supplementation of an Alkylglycerol Mix Comprising Different Alkyl Chains Effectively Modulates Multiple Endogenous Plasmalogen Species in Mice

**DOI:** 10.3390/metabo11050299

**Published:** 2021-05-06

**Authors:** Sudip Paul, Aliki A. Rasmiena, Kevin Huynh, Adam Alexander T. Smith, Natalie A. Mellett, Karin Jandeleit-Dahm, Graeme I. Lancaster, Peter J. Meikle

**Affiliations:** 1 Metabolomics Laboratory, Baker Heart and Diabetes Institute, Melbourne, VIC 3004, Australia; sudip.paul@baker.edu.au (S.P.); aliki.rasmiena@gmail.com (A.A.R.); kevin.huynh@baker.edu.au (K.H.); alexander.smith@baker.edu.au (A.A.T.S.); natalie.mellet@baker.edu.au (N.A.M.); 2 Faculty of Medicine, Nursing and Health Sciences, Monash University, Clayton, VIC 3800, Australia; karin.jandeleit-dahm@monash.edu; 3 Hematopoiesis and Leukocyte Biology Laboratory, Baker Heart and Diabetes Institute, Melbourne, VIC 3004, Australia; graeme.lancaster@baker.edu.au

**Keywords:** plasmalogen, alkylglycerol, ether lipids, metabolic disease

## Abstract

Plasmalogens or alkenylphospholipids are a sub-class of glycerophospholipids with numerous biological functions and are thought to have protective effects against metabolic disease. Dietary supplementation with alkylglycerols (AKGs) has been shown to increase endogenous plasmalogen levels, however effective modulation of different molecular plasmalogen species has not yet been demonstrated. In this study, the effects of an orally-administered AKG mix (a mixture of chimyl, batyl and selachyl alcohol at a 1:1:1 ratio) on plasma and tissue lipids, including plasmalogens, was evaluated. Mice on a Western-type diet were treated with either an AKG mix or vehicle (lecithin) for 1, 2, 4, 8 and 12 weeks. Treatment with the AKG mix significantly increased the total plasmalogen content of plasma, liver and adipose tissue as a result of elevations in multiple plasmalogen species with different alkenyl chains. Alkylphospholipids, the endogenous precursors of plasmalogens, showed a rapid and significant increase in plasma, adipose tissue, liver and skeletal muscle. A significant accumulation of alkyl-diacylglycerol and lyso-ether phospholipids was also observed in plasma and tissues. Additionally, the dynamics of plasmalogen-level changes following AKG mix supplementation differed between tissues. These findings indicate that oral supplementation with an AKG mix is capable of upregulating and maintaining stable expression of multiple molecular plasmalogen species in circulation and tissues.

## 1. Introduction

Plasmalogens are a subclass of glycerophospholipids that consist of an alkenyl chain (generally O-16:0, O-18:0 or O-18:1) at the sn1 position, an acyl chain at the sn2 position, and predominantly a choline or ethanolamine head group at the sn3 position [1]. These specialised phospholipids are important constituents of plasma membranes and are considered as endogenous antioxidant phospholipids due to the vinyl ether linkage at the sn1 position that scavenges reactive oxygen species [2]. Modulation of plasmalogen levels in lipoproteins and phospholipid bilayers has been shown to delay oxidation of low-density lipoprotein and cholesterol [3,4]. Plasmalogens can also play important roles in other cellular processes—they have been shown to be an important regulator of cholesterol biosynthesis [5], and essential for intracellular cholesterol transport [6] and high density lipoprotein-mediated cholesterol efflux [7]. Plasmalogens have been found to be vital for phagocytosis [8] and have anti-inflammatory properties in neuronal and intestinal tract cells [9,10]. They have also recently been identified as mediators of mitochondrial fission in brown adipose tissue and thereby contribute to thermogenesis [11].

Plasmalogens are found in almost all mammalian tissues, however, their abundance is highly variable between tissues; plasmalogen levels are relatively high (≥50% of the glycerophosphoethanolamine fraction) in brain, heart, kidney, skeletal muscle and certain immune cell types, and relatively low (<10% of the glycerophospho-ethanolamine fraction) in liver [12]. The exact reasons for this differential abundance and the tissue-specific function of plasmalogens are unknown.

Plasmalogen deficiency has been observed in various disease conditions. An almost complete deficiency of plasmalogens has been observed in some peroxisomal disorders due to genetic defects affecting the plasmalogen biosynthesis pathway [13]. Reduced levels of plasmalogens have also been reported in complex diseases such as Alzheimer’s disease [14,15], obesity [16], type 2 diabetes [17] and coronary artery disease [18], although a causal link has not been firmly established.

**Figure 1:**
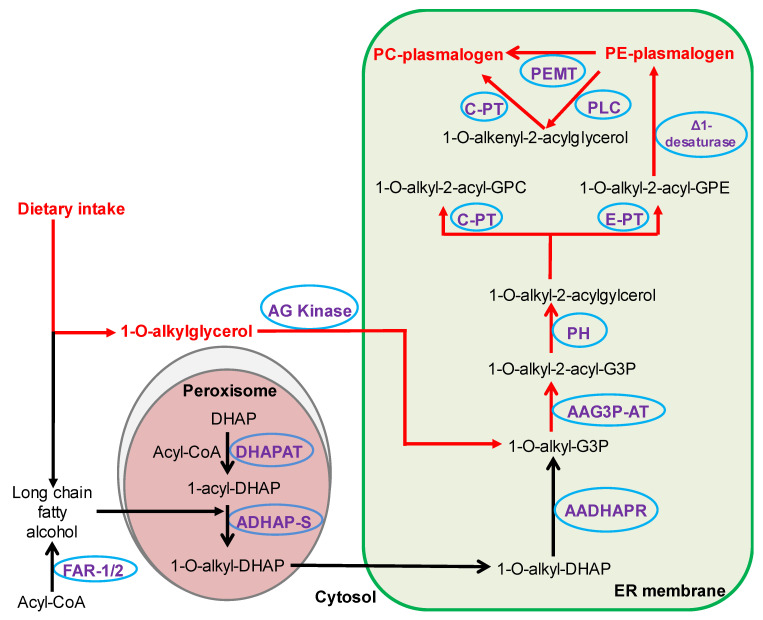
Dietary alkylglycerols can modulate endogenous plasmalogen content. Dietary AKGs can bypass the rate-limiting peroxisomal biosynthetic steps (red pathway). Metabolites are shown in red and black and enzymes are shown in violet. AADHAP-R: alkyl/acyl-DHAP-reductase, AAG3P-AT: alkyl/acyl-glycero-3-phosphate acyltransferase, ADHAP-S: alkyl DHAP synthase, AG kinase: alkylglycerol kinase, CoA: coenzyme A, C-PT: choline phosphotransferase, Δ1-desaturase: plasmanylethanolamine desaturase, DHAP: dihydroxyacetone phosphate, DHAP-AT: DHAP acyltransferase, E-PT: ethanolamine phosphotransferase, FAR-1/2: fatty acyl-CoA reductase 1 or 2, ER: endoplasmic reticulum, GPC: glycerophosphocholine, GPE: glycerophosphoethanolamine; PC: phosphatidylcholine, PE: phosphatidylethanolamine, PEMT: phosphatidylethanolamine N-methyltransferase, PH: phosphohydrolase, PLC: phospholipase C.

To better understand the role of plasmalogens in complex diseases, precursors of plasmalogen biosynthesis have been tested for their potential to modulate plasmalogen levels and influence disease pathogenesis. The most commonly used plasmalogen precursors are alkylglycerols (AKGs). These AKGs can bypass the rate-limiting peroxisomal steps of plasmalogen synthesis and lead to increased levels of endogenous plasmalogens through multiple enzyme-catalysed reactions on the endoplasmic reticulum [19,20,21,22,23,24,25] (Figure 1). However, AKGs have been reported to have distinct effects on molecular plasmalogen species based on their structure. Initially, Das and Hajra [26] showed that supplementation of O-17:0 AKG (20 mg/g of food for 10 days) to young rats led to its incorporation into P-17:0 plasmalogens in different tissues but could not increase the total tissue plasmalogen content. Similar observations were reported by Blank et al. [27], where rats fed with 1-O-alkyl-2,3-diacetyl-sn-glycerol containing 65% O-18:1 and 17% O-16:1 alkyl groups showed an increase in the P-18:1 plasmalogens but no increase in the total plasmalogen content of liver, kidney or lung. In cultured HEp2 cells, supplementation of chimyl alcohol (O-16:0, 20 µM), selectively increased the levels of P-16:0 plasmalogen species [28]. Rasmiena et al. [29] reported that supplementation of batyl alcohol (O-18:0, 2% of dry food weight), significantly increased the circulatory and cardiac plasmalogen content in mice. However, this increase was restricted to the P-18:0 plasmalogen species and there were concurrent decreases in other species (P-16:0 and P-18:1). In a more recent study, batyl alcohol supplementation (1% of dry food weight) also resulted in marked increases in P-18:0 plasmalogen species along with significant decreases in P-16:0 and P-18:1 species in the hearts of mice with cardiac remodeling [30]. Similar to naturally occurring AKGs, a chemically synthesised plasmalogen precursor, PPI-1011 (with O-16:0 alkyl chain at sn1, docosahexaenoic acid at sn2 and lipoic acid at sn3) increased the target plasmalogen (P-16:0/22:6) in plasma but was unable to increase the total circulatory plasmalogen level in mice [31]. Collectively, these studies indicate that modulating plasmalogen levels by supplementation of AKGs with a particular alkyl chain leads to an increase in only plasmalogens containing the corresponding alkenyl chain but can also lead to a compensatory reduction in species containing other alkenyl chains. In the present study, we therefore examined if supplementation with a mix of the major AKGs (chimyl (O-16:0), batyl (O-18:0) and selachyl (O-18:1) alcohols at a 1:1:1 ratio) could increase total plasmalogen levels, while at the same time maintaining the ratios of endogenous plasmalogen species. In addition, we also sought to investigate the global changes in the lipidome resulting from supplementation with an AKG mix.

## 2. Results

### 2.1. Effects of AKG Mix Treatment on Endogenous Alkyl Ether Phospholipids in Mice

AKGs are initially metabolised into alkyl ether phospholipids (PE(O) and PC(O)) and then to alkenyl ether phospholipids or plasmalogens (Figure 1). Hence, an increase in alkyl ether phospholipids following AKG supplementation is a preliminary indicator of the incorporation of AKGs into the plasmalogen biosynthetic pathway.

**Figure 2:**
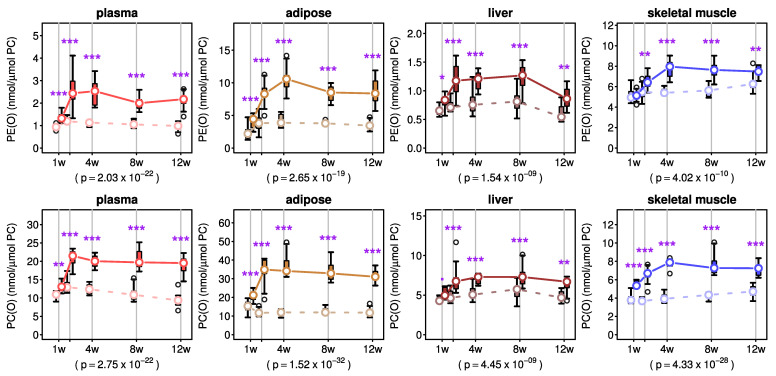
Effects of alkylglycerol mix administration on alkylphospholipid concentrations in plasma and tissues. Concentrations of alkylphosphatidylethanolamine (PE(O)) and alkylphosphatidylcholine (PC(O)) were normalised to total phosphatidylcholine (PC) concentration in plasma and different tissues of C57BL6 mice (*n* = 7–8/group) at each time point (offset for clarity). Treatment (solid lines) and vehicle (dashed lines) groups are shown for each tissue. Boxes show the inter-quartile range and whiskers show the range. Outliers (greater than 1.5 times the inter-quartile range from the median) are shown as empty circles. Medians are shown as white dots and are joined together to facilitate visualisation of the pseudo-kinetics. Post-hoc p values for treatment effect from a two-way ANOVA are also shown. Purple stars indicate significance: * *p* < 0.05, ** *p* < 0.01 and *** *p* < 0.001 from a post-hoc test for the mean differences between treatment and vehicle groups at each time point.

PE(O) and PC(O) levels in plasma, adipose tissue, liver and skeletal muscle progressively increased over the course of AKG supplementation, typically reaching a plateau after 2–4 weeks of AKG treatment, whereupon levels stabilised for the remainder of the 12 week intervention (Figure 2).

A two-way ANOVA between experimental groups showed a significant main effect for AKG treatment without treatment/time interaction on PE(O) concentration in plasma, adipose tissue and liver (Figure 2 and Tables S2–S4). However, in skeletal muscle, there was a significant interaction between treatment and time on PE(O) concentration (Figure 2 and Table S5). Interestingly, there was a significant main effect for time on the PE(O) concentration in plasma and tissues (Figure 2 and Tables S2–S5). In the case of PC(O), a significant interaction effect of treatment and time was observed in plasma, adipose tissue and skeletal muscle while the time effect was significant for plasma, liver and skeletal muscle (Figure 2 and Tables S2–S5). The main AKG treatment effect was significant for plasma and all tissues (Figure 2 and Tables S2–S5).

### 2.2. Effects of AKG Mix Treatment on Endogenous Plasmalogens in Mice

**Figure 3:**
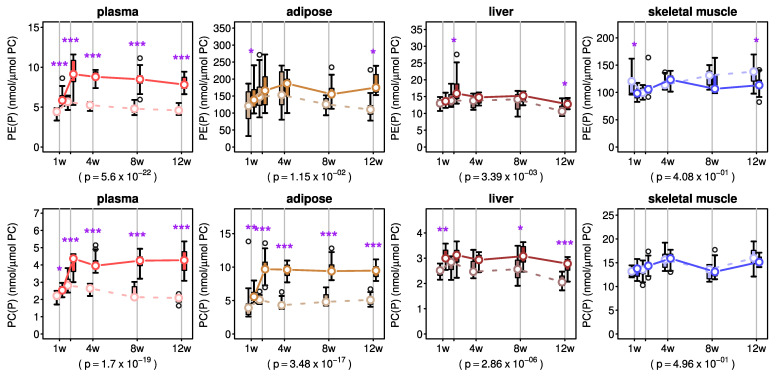
Effects of alkylglycerol mix administration on alkenylphospholipid (plasmalogen) concentrations in plasma and tissues. Concentrations of alkenylphosphatidylethanolamine (PE(P)) and alkenylphosphatidylcholine (PC(P)) were normalised to total phosphatidylcholine (PC) concentration in plasma and different tissues of C57BL6 mice (*n* = 7–8/group) at each time point (offset for clarity). Treatment (solid lines) and vehicle (dashed lines) groups are shown for each tissue. Boxes show the inter-quartile range and whiskers show the range. Outliers (greater than 1.5 times the inter-quartile range from the median) are shown as empty circles. Medians are shown as white dots and are joined together to facilitate visualisation of the pseudo-kinetics. Post-hoc p-values for treatment effect from a two-way ANOVA are also shown. Purple stars indicate significance: * *p* < 0.05, ** *p* < 0.01 and *** *p* < 0.001 from a post-hoc test for the mean differences between treatment and vehicle groups at each time point.

We further looked into the effects of AKG mix treatment on total plasmalogen content of plasma and different tissues. In plasma, the levels of PE(P) and PC(P) progressively increased over the first 2 weeks of AKG mix treatment and this increase was sustained for the duration of 12 weeks intervention (Figure 3). In adipose tissue, the level of PE(P) gradually increased up to 4 weeks of AKG mix treatment and then stabilised (Figure 3). However, in the case of PC(P), the level was almost doubled after only 2 weeks of AKG mix treatment and this augmentation was maintained for the duration of the study (Figure 3). In liver, the increases in PE(P) and PC(P) levels were most prominent after 12 weeks of AKG mix treatment (Figure 3).

A two-way ANOVA revealed a significant main AKG treatment effect without any treatment/time interaction effect on PE(P) level in plasma, adipose tissue or liver (Figure 3 and Supplementary Tables S2–S4). The time main effect on PE(P) level was significant for plasma, liver and skeletal muscle (Figure 3 and Tables S2, S4 and S5). Two-way ANOVA also revealed a significant main effect for AKG treatment and time as well as an interaction effect on PC(P) concentration in plasma (Figure 3 and Table S2). The main effects for treatment and time on PC(P) levels were also significant in adipose tissue and liver (Figure 3 and Tables S3 and S4).

### 2.3. Effects of AKG Mix Treatment on Different Plasmalogen Species in Mice

**Figure 4:**
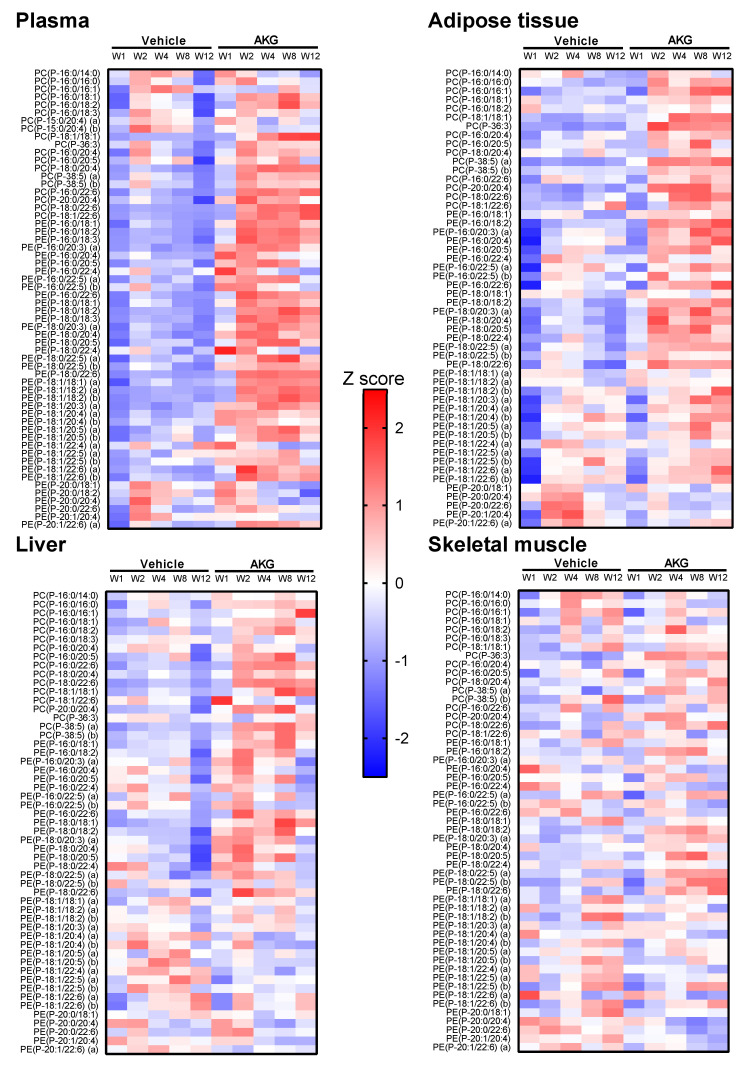
Heatmap showing the effects of alkylglycerol mix administration on plasmalogen species in plasma and tissues. Plasmalogen concentration data are presented as Z scores calculated for all animals in the study (mean-centered and scaled to unit variance per lipid) (by row). W: weeks of treatment, AKG: alkylglycerol mix, PC(P): alkenylphosphatidylcholine; PE(P), alkenylphosphatidylethanolamine.

The endogenous plasmalogen pool is composed of multiple molecular plasmalogen species. To explore the changes in individual plasmalogen species with AKG treatment over time, we built a heat map of plasmalogen concentration Z scores for each tissue. As seen from Figure 4, AKG mix treatment increased the levels of multiple plasma and adipose plasmalogen species containing different alkenyl chains, though only prominently after at least 2 weeks of AKG treatment (Figure 4). The increases in multiple hepatic plasmalogen species were also noticeable but much smaller compared to plasma and adipose tissue (Figure 4). In contrast, there was no specific pattern of changes in skeletal muscle plasmalogen species over time in both vehicle and AKG treatment groups (Figure 4). Two-way ANOVA also revealed that there were significant main AKG treatment effects for multiple plasmalogen species in plasma and other tissues (Tables S6–S9).

**Figure 5:**
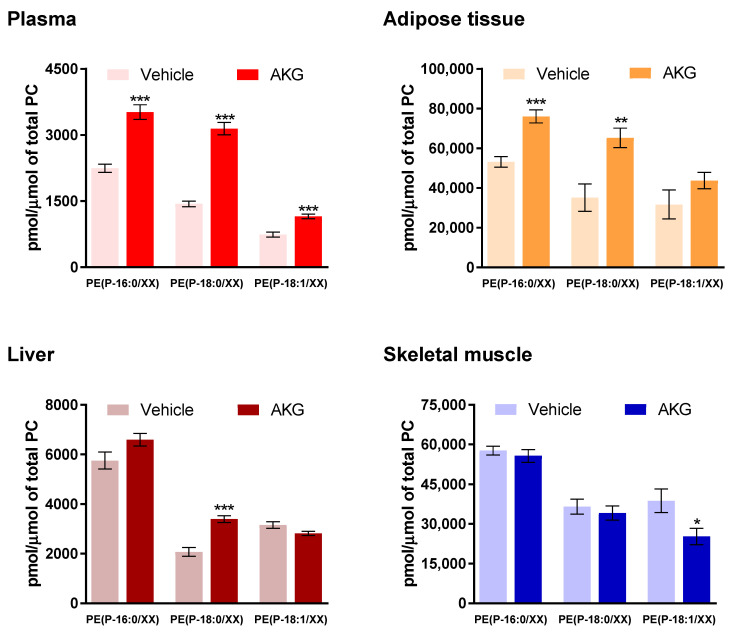
Effects of alkylglycerol mix administration on endogenous plasmalogens with different alkenyl chains. Concentrations of alkenylphosphatidylethanolamine (PE(P)) with different alkenyl chains were normalised to total phosphatidylcholine (PC) concentration in plasma and different tissues of C57BL6 mice at the 12-week time point. Data are presented as mean ± SEM (*n* = 8/group). Treatment (darker colours) and vehicle (lighter colours) groups are shown for each tissue. The mean differences between two dietary groups (vehicle and treatment) were analysed using Student’s *t*-test; * indicates *p* < 0.05, ** indicates *p* < 0.01 and *** indicates *p* < 0.001 relative to the vehicle group.

For a clearer understanding of the effects of AKG mix treatment on alkenyl chain composition of endogenous plasmalogens, we grouped PE plasmalogen species based on their alkenyl chain (PE(P-16:0), PE(P-18:0) and PE(P-18:1)) and then compared the levels between AKG mix and vehicle treatment groups. For this analysis we selected PE plasmalogen species because the alkenyl chain composition of these species was precisely identified. We observed that AKG mix treatment significantly increased plasma PE plasmalogens with different alkenyl chains (P-16:0, P-18:0 and P-18:1) in plasma (Figure 5), however the magnitude of increase was different for different alkenyl sub-groups. Indeed, the highest increase was observed for PE(P-18:0) plasmalogens and the lowest for PE(P-18:1) plasmalogens (Figure 5). In adipose tissue, AKG mix treatment also increased PE plasmalogens with different alkenyl chains, however, the increases in PE(P-16:0) and PE(P-18:0) plasmalogens with AKG mix treatment were statistically significant and the increase in PE(P-18:1) plasmalogens was non-significant (Figure 5). In the liver, there was a significant increase in PE(P-18:0) plasmalogen species and a non-significant increase in PE(P-16:0) plasmalogen species following AKG mix treatment (Figure 5). In skeletal muscle, there was no significant change in PE(P-16:0) and PE(P-18:0) plasmalogens, but a significant reduction in PE(P-18:1) plasmalogens with AKG mix treatment (Figure 5).

### 2.4. Effects of AKG Mix Treatment on Ether Triacylglycerols and Lyso-Ether Phospholipids in Mice

**Figure 6:**
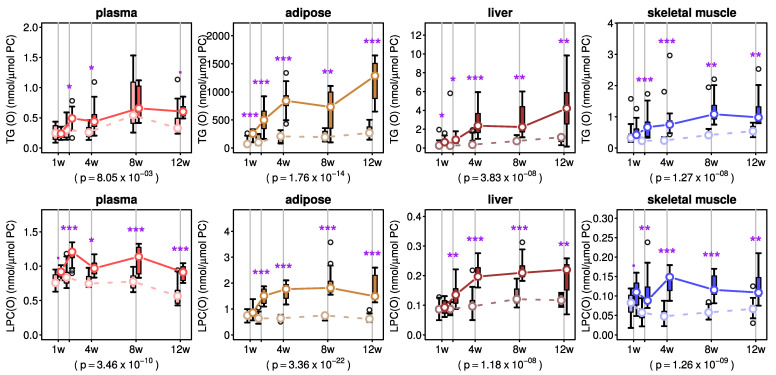
Effects of alkylglycerol mix administration on ether triacylglycerol and lyso-alkylphosphatidylcholine concentrations in plasma and different tissues. Concentrations of ether triacylglycerol or monoalkyl-diacylglycerol or TG(O) and lyso-alkylphosphatidylcholine or LPC(O) were normalised to total phosphatidylcholine (PC) concentration in plasma and different tissues of C57BL6 mice (*n* = 7–8/group) at each time point (offset for clarity). Treatment (solid lines) and vehicle (dashed lines) groups are shown for each tissue. Boxes show the inter-quartile range and whiskers show the range. Outliers (greater than 1.5 times the inter-quartile range from the median) are shown as empty circles. Medians are shown as white dots and are joined together to facilitate visualisation of the pseudo-kinetics. Post-hoc p values for treatment effect from a two-way ANOVA are also shown. Purple stars indicate significance: * *p* < 0.05, ** *p* < 0.01 and *** *p* < 0.001 from a post-hoc test for the mean differences between treatment and vehicle groups at each time point.

Monoalkyl-diacylglycerols, also referred to as ether triacylglycerols (TG(O)), are similar to common triacylglycerols (TG), with the exception that they contain an O-alkyl chain at the sn1 position instead of an acyl chain [32,33]. In this study, we measured 18 different TG(O) species in plasma and tissues to assess how AKG mix treatment affects this pool of neutral ether lipids. As seen in Figure 6, TG(O) concentration of plasma and tissues steadily increased over the 4 weeks of AKG treatment and then persisted for the duration of the study. Two-way ANOVA did not show any significant interaction between treatment and time on TG(O) concentration in plasma and tissues. Instead, main effects were significant for treatment and time (Figure 6 and Tables S2–S5).

We also explored the effects of AKG mix treatment on the lyso forms of ether phospholipids (LPC(O) and LPE(P)). In plasma, although the LPC(O) levels of AKG-treated mice fluctuated throughout the experimental period, they were significantly higher than in vehicle-treated mice at 2 weeks and later time points (Figure 6). In adipose tissue and liver, LPC(O) level gradually increased over the first 4 weeks of AKG mix treatment and remained stable for the duration of the study (Figure 6). In skeletal muscle, there was a stable increase in LPC(O) level after 4 weeks of AKG mix treatment, which was reasonably maintained up to 12 weeks (Figure 6). Two-way ANOVA showed that there was no significant interaction between treatment and time on LPC(O) level in plasma and tissues except adipose tissue (Figure 6 and Supplementary Tables S2–S5). The main time effect was significant for plasma, adipose tissue and liver (Figure 6 and Tables S2–S4). However, the main AKG treatment effect was significant for plasma and all tissues (Figure 6 and Tables S2–S5).

We also observed a significant main AKG treatment effect on LPE(P) level in plasma, adipose tissue and liver without treatment/time interaction, however, the changes in LPE(P) level were variable throughout the experimental period (Tables S2–S4).

### 2.5. Effects of Alkylglycerol Mix Treatment on Sphingolipids, Lysophospholipids, Phospholipids and Neutral Lipids in Mice

We also analysed the effects of AKG mix administration on lipid classes besides ether phospholipids. In plasma, there was an overall significant decrease in free cholesterol levels with AKG mix treatment (Table S2). In adipose tissue, there was an overall significant increase in sphingomyelin (SM) content with AKG mix treatment (Table S3). An overall decrease in hepatic phosphatidylglycerol (PG) content was also observed with AKG mix treatment (Table S4).

## 3. Discussion

The ability of AKGs to modulate endogenous plasmalogen levels has been demonstrated in several studies [26,27,28,29,30,33]. However, the optimal formulation of AKG species for the modulation of endogenous plasmalogens has not been defined. The need for such a formulation has arisen from the observation that supplementation with an individual AKG (e.g., O-18:0 alone) only increases the corresponding plasmalogen species (e.g., P-18:0), and often results in a decrease in other plasmalogen species (e.g., P-16:0 and P-18:1). An optimal formulation of AKGs may be required to maintain the endogenous plasmalogen composition, particularly with respect to the sn1 alkenyl groups, and this in turn may be important in obtaining maximum therapeutic benefits from plasmalogen modulation. Hence, in the present study, we examined whether supplementation of a combination of AKGs can lead to an effective, balanced modulation of the multiple endogenous plasmalogen species.

In plasma, we observed a rapid increase in both PE and PC plasmalogen levels with AKG mix treatment, which was maintained for the duration of the study. The increase in plasma plasmalogens was due to marked elevations in multiple plasmalogen species containing different alkenyl chains (P-16:0, P-18:0 and P-18:1). It is however important to note that the supplementation of an equimolar mixture of major AKGs did not increase the corresponding plasmalogens in the same magnitude. We also noted that the changes in plasma lipids following AKG mix treatment was mostly confined to plasmalogens or related ether lipids.

Previously, Das and Hajra [26] showed that about 50% of dietary AKGs are absorbed intact and transported to different tissues, where they can be incorporated into endogenous ether lipids including plasmalogens. Here, we performed lipidomic analysis on adipose tissue, liver and skeletal muscle to assess the effects of AKG mix treatment on the accumulation of plasmalogens in these tissues. Our results suggest that, in adipose tissue, the AKGs were rapidly incorporated into alkyl ether phospholipids (PE(O) and PC(O)), the first sign of their entrance into the plasmalogen biosynthesis pathway. Similar to plasma, we also observed a significant increase in total adipose tissue plasmalogen content with AKG mix treatment, which was due to increases in multiple plasmalogen species with different alkenyl chains. However, in adipose tissue, the magnitude of increase in PC plasmalogen content was much higher than PE plasmalogen content. Moreover, in this tissue, the increase in PC plasmalogen was much more rapid than PE plasmalogen after AKG mix treatment. These differential responses may be due to very active conversion of PE(P) to PC(P) and/or differential regulation and turnover of these plasmalogen sub-types in this tissue. In adipose tissue, we also observed a rapid and marked increase in the LPC(O) level after AKG mix treatment. This could result from an accelerated conversion of PC(O) into LPC(O) by the phospholipase A2 (PLA2) enzyme. In addition to the changes in ether phospholipids, we also observed a significant increase in TG(O) content of adipose tissue following AKG mix treatment. The biological function of TG(O) in mammalian tissues is not yet known, except for their speculated role in the transportation of AKGs from intestine to different organs [33]. Our findings suggest that the AKGs are rapidly and actively esterified into these TG(O) species for storage in the adipose tissue. This pool of neutral ether lipids could be utilised to produce ether phospholipids when needed after partial or complete deacylation.

Similar to adipose tissue, we observed that AKGs were also rapidly fluxed into ether phospholipids (PE(O) and PC(O)) in the liver. In liver, we also observed a significant increase of total plasmalogen content following AKG mix treatment. Here, the increases in P-18:0 plasmalogen species were more prominent than other alkenyl-chain-containing species. It is possible that the liver produces high amounts of plasmalogens utilising the AKGs but rapidly releases them into the circulation via lipoproteins, thus reflecting a smaller net change in liver plasmalogen levels compared to plasma or adipose tissue. Alternately, the enzyme responsible for PE(P) synthesis from PE(O) may be inactive in the liver. High expression of the AKG-catabolising enzyme, a so-called AKG-monooxygenase (AGMO or TMEM195; EC 1.14.16.5) that cleaves AKGs into glycerol and fatty aldehyde in the liver [34] may also contribute to the relatively small increase in hepatic plasmalogens. Similar to adipose tissue, there was a significant accumulation of LPC(O) and TG(O) with AKG mix treatment in the liver.

In contrast to plasma, adipose tissue and liver, we did not observe any increase in the plasmalogen levels of skeletal muscle with AKG mix treatment. However, there was a significant increase in ether phospholipid levels (both PE(O) and PC(O)) of skeletal muscle after AKG mix treatment. These findings imply that the AKGs were shuttled into the plasmalogen biosynthetic pathway in this tissue, but the levels of plasmalogens might be more tightly regulated, preventing an overall increase in plasmalogen levels. This regulation could occur at the conversion of PE(O) to PE(P) step, mediated by the enzyme plasmanylethanolamine desaturase. The gene that encodes this enzyme, *Tmem189* has recently been identified by Gallego-Garcia et al. [23]. They also reported that *Tmem189*-deficient HAP1 cells do not have any plasmalogens but have higher ether phospholipids than wild-type cells. The function of this enzyme in plasmalogen biosynthesis has been further elucidated by Werner et al. [35]. However, the role of this enzyme in regulating the levels of endogenous plasmalogens under different physiological and pathological conditions should be investigated further. In our study, significant accumulations of LPC(O) and TG(O) with AKG mix treatment also support the notion that AKGs were actively distributed into ether lipids but were not capable of increasing plasmalogens due to a strict regulation of plasmalogen levels in skeletal muscle. However, such ether lipid species may act as a reserve in skeletal muscle and could be utilised to produce plasmalogens in times of need, such as oxidative stress, via activating the plasmanylethanolamine desaturase enzyme.

Our findings suggest that AKG mix treatment led to an increase of endogenous plasmalogen species while maintaining the endogenous composition of the alkenyl chains. The results also indicate that the rate and capacity of AKG flux into plasmalogens are distinct in different tissues. This could be due to different regulatory mechanisms operating in various tissues; however, further studies are warranted to elucidate these mechanisms. This study has several limitations; first, we only tested one dose of the alkylglycerol mix, second, we characterised the lipidomes of selective tissues and third, we didn’t assess the therapeutic benefits of using AKG mix instead of an individual AKG. Despite these limitations, our data provide valuable information about the differential changes in circulatory and tissue plasmalogens with AKG mix supplementation, in particular the increases in plasma and adipose tissue plasmalogens are quite striking. Considering the effective enrichment of endogenous plasmalogens by AKG mix supplementation, this supplement should now be further tested for its ability in ameliorating clinical complications in diseases where plasmalogen deficiency has been identified but a causal relationship has not been clearly established.

## 4. Materials and Methods

### 4.1. Preparation of the AKG Mix

To facilitate the administration of the AKG mix, lecithin was used as a vehicle. Stock solutions of individual AKGs (100 mg/mL; batyl and chimyl alcohols (chirally pure), Bachem, Bubendorf, Switzerland, and selachyl alcohol (racemic mixture), Astral Scientific, Sydney, NSW, Australia) were firstly prepared in chloroform: methanol (1:1). A stock solution of lecithin (500 mg/mL; Sigma-Aldrich, Saint Louis, MO, USA) was also prepared in chloroform:methanol (1:1). To prepare the AKG mix, 1.0 mL of each of the AKG stock solutions were mixed, 500 µL of lecithin stock solution was added, dried under a stream of nitrogen gas at 40 °C and then reconstituted in 5 mL of deionised water by sonication in an Ultrasonic Cleaner water bath (Soniclean, Adelaide, SA, Australia) for 1–2 h and then further sonicated using a Misonix S-4000 Sonicator (Thermo Fisher Scientific, Melbourne, VIC, Australia) for 3 × 30 s at amplitude 25. A vehicle control was also prepared. For this, 500 µL of lecithin stock solution was dried and reconstituted with deionised water as described above.

### 4.2. Animal Experimentation

Eight-week-old male C57BL/6 mice (Alfred Medical Research and Education Precinct, Melbourne, VIC, Australia) were fed a Western-type diet (22% fat, 0.15% cholesterol; SF00-219, Specialty Feeds, Glen Forrest, WA, Australia). The animals were housed in standard conditions with unrestricted access to food and water at the Precinct Animal Centre of the Baker Heart and Diabetes Institute. They were maintained on a 12 h light and dark cycle in a pathogen-free environment. They received a daily dose of 10 mg of vehicle (lecithin) or 12 mg of AKG mix (batyl alcohol:chimyl alcohol:selachyl alcohol = 1:1:1) plus 10 mg of vehicle in 200 µL of deionised water, via oral gavage for 1, 2, 4, 8 or 12 weeks (*n* = 7–8/group). After the treatment period, animals were anaesthetised by intraperitoneal injection of Avertin (2,2,2-tribromoethanol) (0.3 mL of 2.5% solution per 20 g mouse; Sigma-Aldrich, Saint Louis, MO, USA) following food withdrawal for 5 h, and organs were rapidly dissected and snap frozen. During the dissection, whole blood was collected by direct puncture of the left ventricle into EDTA tubes. Plasma was separated from the blood via centrifugation (1485× *g*, 10 min, room temperature).

The experiment was approved and conducted in accordance with the principles devised by the Alfred Medical Research and Education Precinct Animal Ethics Committee (E/1503/2014/B) under guidelines laid down by the National Health and Medical Research of Council of Australia.

### 4.3. Tissue Homogenisation

Approximately 30–60 mg of tissues were homogenised in 500 µL of ice-cold phosphate buffered saline (pH 7.6) using a Bio-Gen Pro200 electric homogeniser (PRO Scientific, Oxford, CT, USA) for 10 s and then sonicated with a Misonix S-4000 Sonicator (Thermo Fisher Scientific, Melbourne, VIC, Australia) for 15 s at amplitude 20. Protein content of the homogenates was quantified using a Pierce^TM^ BCA protein assay kit (Thermo Fisher Scientific, Rockford, IL, USA). Homogenates were then made up to a stock protein concentration of 5 mg/mL protein and 10 µL aliquots from the stock solutions, containing 50 μg of protein, were subsequently used for lipid extraction.

### 4.4. Lipid Extraction

Prior to lipid extraction, samples were randomised to reduce bias. Lipids were extracted as described previously [18]. Briefly, plasma or homogenised tissue was combined with internal standard mix (Table S1) and the lipids were extracted using 20 volumes of chloroform:methanol (2:1). The extracted lipids were dried under a stream of nitrogen at 40 °C and subsequently reconstituted in 1:1 mixture of water saturated butanol and methanol containing 10 mmol/L ammonium formate.

### 4.5. Liquid Chromatography and Electrospray Ionisation Tandem Mass Spectrometry

Lipids were quantified using multiple reaction monitoring mode on an Agilent 1200 high pressure liquid chromatography system coupled to a Q/TRAP 4000 triple quadrupole mass spectrometer (AB SCIEX) using methodology described previously [36]. Liquid chromatography separation was performed on a 2.1 × 100 mm C18 Poroshell column (Agilent, Santa Clara, CA, USA) at 400 µL/min. The following gradient conditions were used: 10% B to 55% B over 3 min, then to 70% B over 8 min, to 89% B over 0.1 min, and finally to 100% B over 3.3 min. The solvent was then held at 100% B for 1 min. Equilibration was as follows: solvent was decreased from 100% B to 10% B over 0.1 min and held for an additional 4.5 min. The solvent system consisted of solvent A: 50% H_2_O/30% acetonitrile/20% isopropanol (*v*/*v*/*v*) containing 10 mM ammonium formate and solvent B: 1% H_2_O/9% acetonitrile/90% isopropanol (*v*/*v*/*v*) containing 10 mM ammonium formate. The conditions for the tandem mass spectrometry of each lipid class are provided in Table S1. Lipid species within each class were analysed the same way. The levels of individual lipid species were calculated by taking a ratio of the area under the curve of the lipid of interest to the area under the curve of corresponding internal standard. The ratio was then multiplied by the amount of internal standard added into the sample. Response factors were applied to some lipid species to improve accuracy of the measurements as described previously [16]. The levels for lipid classes were calculated from the sum of individual species within each class.

### 4.6. Statistical Analysis

The lipidomic data were normalised to the level of total phosphatidylcholine (the major phospholipid class) in plasma and different tissues to allow a direct comparison of the relative lipid levels between the sample types and were thus expressed as either pmol/µmol total PC or nmol/µmol total PC. Prior to normalisation, we compared the levels of total PC (normalised to volume and protein for plasma and tissues respectively) between vehicle and AKG treatment groups at different time points by Student’s *t*-test to check whether there was any effect of AKG treatment on total PC level. We did not observe any significant difference in total PC level between the vehicle and AKG treatment groups. A two-way ANOVA was used to analyse the effects of treatment and time and their interaction on lipid concentration in plasma and tissues. This model was interrogated using appropriate contrasts to compare the mean levels of vehicle and AKG treatment groups at each time point. For PE(P) alkenyl chain composition analysis, the data from the 12 week time point were used and the mean differences between the vehicle and AKG treatment groups were compared by a Student’s *t*-test. Obtained p-values were corrected by the Benjamini–Hochberg method, and values less than 0.05 were considered statistically significant. All statistical analyses were carried out in R (×64, Version 3.5.0) (R Foundation for Statistical Computing, Vienna, Austria).

## 5. Conclusions

For the first time, we demonstrated that a wide range of circulatory and tissue plasmalogens comprising different alkenyl chains can be increased by supplementation of a mixture of different AKGs. This unique plasmalogen modulation approach could provide better therapeutic benefits than the existing approaches in multiple disease settings.

## Data Availability

The data presented in this manuscript are available on request from the corresponding author.

## Supplementary Materials

The following are available online at https://www.mdpi.com/article/10.3390/metabo11050299/s1, Table S1: Internal standards and mass spectrometry conditions used for lipid analysis in this study, Table S2: Effects of alkylglycerol mix administration on plasma lipid classes in mice, Table S3: Effects of alkylglycerol mix administration on adipose tissue lipid classes in mice, Table S4: Effects of alkylglycerol mix administration on hepatic lipid classes in mice, Table S5: Effects of alkylglycerol mix administration on skeletal muscle lipid classes in mice, Table S6: Effects of alkylglycerol mix administration on plasma lipid species in mice, Table S7: Effects of alkylglycerol mix administration on adipose tissue lipid species in mice, Table S8: Effects of alkylglycerol mix administration on hepatic lipid species in mice, Table S9: Effects of alkylglycerol mix administration on skeletal muscle lipid species in mice.
